# Identification of Hub Genes Involved in Early-onset Schizophrenia: From Genetic Susceptibility to Predicted Regulated Gene Expression

**DOI:** 10.21203/rs.3.rs-5833160/v1

**Published:** 2025-01-20

**Authors:** Yawen Jen, Sung-Liang Yu, Po-Chang Hsiao, Po-Hsiu Kuo, Chih-Min Liu, Chen-Chung Liu, Tzung-Jeng Hwang, Ming H. Hsieh, Yi-Ling Chien, Yi-Ting Lin, Hailiang Huang, Yen-Chen Anne Feng, Chuhsing K. Hsiao, Yen-Feng Lin, Stephen V. Faraone, Benjamin Neale, Stephen J. Glatt, Ming T. Tsuang, Hai-Gwo Hwu, Wei J. Chen

**Affiliations:** Center for Neuropsychiatric Research, National Health Research Institutes; Department of Clinical Laboratory Sciences and Medical Biotechnology, College of Medicine, National Taiwan University; Institute of Epidemiology and Preventive Medicine, College of Public Health, National Taiwan University; Institute of Epidemiology and Preventive Medicine, College of Public Health, National Taiwan University; National Taiwan University Hospital; National Taiwan University Hospital; National Taiwan University Hospital; National Taiwan University Hospital; National Taiwan University Hospital; National Taiwan University Hospital; Stanley Center for Psychiatric Research, Broad Institute of MIT and Harvard; Institute of Health Data Analytics and Statistics, College of Public Health, National Taiwan University; Institute of Epidemiology and Preventive Medicine, College of Public Health, National Taiwan University; Center for Neuropsychiatric Research, National Health Research Institutes; Departments of Psychiatry and Behavioral Sciences, Neuroscience and Physiology, and Public Health and Preventive Medicine, SUNY Upstate Medical University; Stanley Center for Psychiatric Research, Broad Institute of MIT and Harvard; Departments of Psychiatry and Behavioral Sciences, Neuroscience and Physiology, and Public Health and Preventive Medicine, SUNY Upstate Medical University; Center for Behavioral Genomics, Department of Psychiatry, University of California San Diego; National Taiwan University Hospital; Center for Neuropsychiatric Research, National Health Research Institutes

**Keywords:** Schizophrenia, Polygenic risk score, Gene expression risk scores, Early onset, Coexpression analyses, Network analysis

## Abstract

**BACKGROUND:**

Despite a high heritability of schizophrenia (SZ), only limited variance was attributed to gene loci or the polygenic risk score in genome-wide association studies (GWAS). Early-onset SZ, a more homogeneous SZ subtype, may aid in bridging the genotype-phenotype gap and the identification of its hub genes is critical for early intervention in clinical practice. We aimed to examine the gene expression risk score (GeRS) in patients from both multiplex and simplex families to identify hub genes for early-onset SZ, and perform enrichment analysis to understand the biological functions of the hub genes.

**METHODS:**

Based on the GWAS genotype data from patients with SZ in multiplex families (223 early-onset and 372 late-onset) and those from simplex families (matched for sex and onset age), GeRSs for SZ (SZ-GeRSs) were estimated using the SNP-expression prediction model derived from existing brain tissues of patients with psychiatric disorders. Module-based SZ-GeRS was summed over genes from empirically derived gene clusters, network analysis was conducted to identify hub genes, and enrichment analysis was used for functional mapping.

**RESULTS:**

Among the 13 modules from existing coexpression analyses of *postmortem* brains of patients with psychiatric disorders, the meta-analysis revealed that associations with early-onset SZ existed for the GeRS of module 10 in subset, M10_sub_-GeRS (adjusted odds ratio [aOR] = 1.38, 95% CI = 1.22–1.57), and six hub genes, M10_hub_-GeRS (aOR = 1.22, 95% CI = 1.07–1.39), after adjustment for covariates. Functional mapping of the genes revealed their enrichment in excitatory neurons and immune-regulatory pathways.

**CONCLUSIONS:**

GeRS for SZ helps identify six hub genes for early-onset schizophrenia, and the enrichment analysis sheds light on their possible roles in the pathophysiology. These findings will enhance the understanding of SZ etiology and may contribute to early screening and personalized prevention efforts.

## Introduction

Schizophrenia (SZ) is a leading cause of disability worldwide (1), with a median lifetime prevalence of 6.35 per 1000 people (2). Despite a heritability of 80% (3, 4), only limited variance in SZ was attributed to gene loci or the polygenic risk score (PRS) in genome-wide association studies (GWASs) (5–7). SZ is a heterogeneous disorder characterized by psychotic symptoms (delusions and hallucinations) and cognitive impairments (8). Given that a substantial proportion of the heritability of SZ remains unexplained, varying levels of gene expression resulting from the interaction of genes and environmental factors are postulated to further contribute to the occurrence of SZ, although collecting *postmortem* brain tissues for gene expression analysis is challenging (9, 10). Instead, predicting genetically regulated gene expression in the brain by integrating an existing tissue-specific single-nucleotide polymorphism (SNP)-expression prediction model with GWAS-based genotype data, i.e., transcriptome imputation, is an alternative approach (11, 12). Studies applying the genetically regulated gene expression risk score (GeRS) for SZ have revealed their associations with SZ or SZ-related traits, including clinical symptomatology and patients’ cognitive performance (12–15). However, only a few genes with potential biological mechanisms that could aid in clinical implementation have been identified in these studies.

Early-onset SZ, occurring in about 8.2% of cases diagnosed before age 18 (16), is a distinct and homogeneous subtype of SZ that serves as a critical phenotype for dissecting its genetic etiology. This subtype is associated with more severe symptoms (17), a greater genetic burden (18, 19), and a higher risk of treatment resistance (20) compared to adult-onset SZ. Findings from GWAS for SZ have identified genetic loci of suggestive significance in early-onset SZ (21, 22), highlighting its potential as a phenotype for uncovering genes relevant to early intervention. However, the genetic basis of early-onset SZ remains largely unknown (23). To elucidate the genetic underpinnings of early-onset SZ, we aimed to examine the gene expression risk score (GeRS) in patients from both multiplex and simplex families to identify hub genes for early-onset SZ, and perform enrichment analysis to understand the biological functions of the hub genes.

## Methods And Materials

### Study workflow

The study workflow is shown in **Additional file 1: Figure S1**. We first predicted genetically regulated gene expression levels in the dorsolateral prefrontal cortex (DLPFC) using the PrediXcan (https://github.com/hakyimlab/PrediXcan), based on the GWAS genotype data from patients with SZ in multiplex families. The SNP-expression prediction model was based on the CommonMind Consortium (CMC; including 254 SZ, 52 bipolar disorder, and 279 controls) (https://predictdb.org/) (12). To extract disease-associated gene expression changes, we then performed a transcriptome-wide association study (TWAS) applying S-PrediXcan (24) with the same SNP-expression prediction model and the latest GWAS summary statistics from the SZ cohort of the Psychiatric Genomics Consortium (PGC; 76,755 patients with SZ and 243,648 controls) (7). After that, we calculated GeRS for each gene by weighting its predicted expression based on the corresponding effect size from the TWAS for SZ. Furthermore, we constructed the module-based GeRS by summing the GeRS of genes listed in the modules obtained from the published weighted gene coexpression network analysis (WGCNA) results (participants including 150 SZ, 94 bipolar disorder, 87 major depression, 50 autism, 17 alcoholism, and 293 controls) (25). Finally, we evaluated the relationship between the module-based GeRS and early-onset SZ in the multiplex sample and explored the hub genes within the early-onset-related module using Ingenuity Pathway Analysis (IPA^®^, QIAGEN Redwood City, CA, USA; www.qiagen.com/ingenuity). We then repeated the analysis in the simplex sample of patients with SZ from trio families.

### Participants

This study included two samples: a multiplex sample and a simplex sample. The multiplex sample was derived from the Taiwan Schizophrenia Linkage Study (TSLS), which recruited Han Chinese families with more than two siblings affected by SZ and their first-degree relatives across Taiwan from 1998–2002 (26, 27) (see details in the **Additional file 1**). After quality control, 595 patients and 570 unaffected relatives from 314 families, with age-at-onset information for the first psychotic episode obtained via the Diagnostic Interview for Genetic Studies (DIGS) (28), were included.

To examine the findings derived from the multiplex sample in patients with different genetic loadings, i.e., the simplex sample, we also included patients who had individual genotype data and age at onset information from another independent sample, the Schizophrenia Trio Genomic Research in Taiwan (S-TOGET). Among the 2,923 patients with SZ from simplex families in S-TOGET, 1,649 had genotype data, and 1,635 of them also had information on age at onset. More detailed information about the S-TOGET cohort has been described previously (29, 30).

To minimize sociodemographic differences between the TSLS and S-TOGET samples, sex- and onset-age-matched subsamples were selected for comparable analysis (**Additional file 1: Table S1**). Both studies were approved by the Institutional Review Board of National Taiwan University Hospital, and all participants provided written informed consent (see details in the **Additional file 1**).

### Measurements

The age at onset and basic sociodemographic characteristics were extracted from the DIGS, with the former obtained from the psychosis section that inquired about the age at onset of the first psychotic episode. We classified age at onset as binary, defining early-onset SZ as psychotic symptoms emerging before age 18, based on our prior research (30).

Furthermore, cognitive function was assessed using the Continuous Performance Test (CPT) for sustained attention (31) and the Wisconsin Card Sorting Test (WCST) for executive function (32). CPT sensitivity index (d′), WCST perseverative errors, and categories achieved were included as cognitive indices. Further details on measurements are provided in the **Additional file 1**.

### Genotyping, quality control, and imputation

Genotyping for the multiplex and simplex samples was performed using the PsychChip array version 1.1 (Illumina, San Diego, CA), containing 588,628 SNPs. Standard quality control (QC) procedures (33) were followed (see details in the **Additional file 1**).

For the multiplex sample, 1,224 participants and 266,899 SNPs that passed QC were used for genotype imputation, and principal components (PCs) were calculated to account for ancestry in subsequent analyses. The genomic inflation factor (λ) of this final sample was 1.07, and the corresponding QQ plot is shown in **Additional file 1:Figure S2.** After genotype imputation, the post-imputation QC filtered for the variants included poor imputation quality (INFO score < 0.8) and low minor allele frequency (MAF < 0.1). Ultimately, 4,853,692 SNPs were included in later analyses.

For the simplex sample, the same QC procedures resulted in 293,643 SNPs that passed QC, with imputation yielding 5,154,665 SNPs. Details of the QC process have been described previously (29, 30).

### Calculation of PRS

PRSs for schizophrenia (SZ-PRS) were calculated for both samples using the p-value thresholding method, utilizing the latest version of summary statistics from the PGC SZ cohort (7). All the SZ-PRS were normalized to z scores for easier interpretation. The best-performing p-value threshold for the SZ-PRS, which explained the most variance in early-onset SZ, was selected for further analysis. Additional details are available in the **Additional file 1**.

### Deriving the effect sizes of predicted gene expression

The tissue-specific TWAS for SZ was performed by S-PrediXcan (24), which integrates GWAS summary data of patients with SZ with a SNP-expression prediction model to identify genes associated with SZ (see see details in the **Additional file 1**). The existing SNP-expression prediction model from CMC (12), which was derived from DLPFC brain tissue samples, was used to perform the TWAS for SZ.

### Predicting gene expression using individual genotypes

To determine the genetically regulated expression predicted for a SNP, hereafter referred to as predicted gene expression, we used PrediXcan (11) to incorporate the genotypes of individual patients and the corresponding SNP-expression prediction model. The DLPFC SNP-expression prediction model from CMC was used to predict gene expression, and only those genotypes that passed standard quality control procedures were subjected to such prediction.

### Calculation of SZ-GeRS

For each gene, its predicted SZ-GeRS was calculated by weighting the predicted gene expression for each patient using the effect size from the TWAS for SZ described in the preceding section. In addition to the SZ-GeRS of individual genes, we constructed two other types of SZ-GeRS by summing genes to identify biologically meaningful relationships with early-onset SZ.

The first type of gene summary SZ-GeRS was a module-based SZ-GeRS, where we utilized gene lists from 13 coexpression modules related to psychiatric disorders identified in a previous WGCNA (25). These modules were derived from postmortem brain tissues of patients with psychotic disorders compared with healthy controls, and we summed the individual SZ-GeRS over the genes listed in those modules. The other type of gene summary SZ-GeRS was constructed by summing over the hub genes of a module. Hub genes were identified via network analysis using IPA, selecting genes regulating the expression of four or more other genes in the top-ranking network. All the SZ-GeRS results were then normalized to z scores for easy interpretation.

Furthermore, we utilized the gene lists from the newly derived coexpression modules identified by the same research group using narrowed samples (32) to recalculate the module-based GeRS and assess the reproducibility of the early-onset-related module and its hub genes identified in these procedures (see details in the **Additional file 1**).

### Enrichment analyses

To understand possible biological functions of the hub genes, we conducted enrichment analyses using the GENE2FUNC function from the functional mapping and annotation platform (FUMA, https://fuma.ctglab.nl/) to annotate them in the biological context. The gene expression datasets from the BrainSpin consisted of brain tissue samples across various developmental stages. We used the Molecular Signatures Database (MsigDB) (34) to assess enrichment in chemical and genetic perturbation gene sets.

Additionally, cell type enrichment analysis was conducted using the PsychSCREEN gene portal (https://psychscreen.beta.wenglab.org/psychscreen/gene), designed to explore single-cell expression of genes in psychiatric disease. The Schizophrenia Bipolar Disorder Multi-Omics Sequencing dataset from PsychENCODE was utilized for this analysis.

### Statistical analyses

Associations between risk scores (including SZ-PRS and all types of predicted SZ-GeRS) and early-onset SZ were assessed using a mixed-effects logistic regression model for the multiplex sample, with family index as a random effect. Covariates included sex, education level, and the first four ancestry PCs. For the simplex sample, a logistic regression model was used with identical covariates. The proportion of explained variance was estimated by calculating the change in Nagelkerke’s pseudo-R^2^ between the null model (containing only the covariates) and the full model (containing covariates + risk scores) (35). We then conducted a meta-analysis of both samples using the R package “metafor” (36), employing a restricted maximum likelihood estimator with inverse-variance weighting.

The SZ-PRS and PCs were computed using the PLINK (37), whereas regression analyses and Nagelkerke’s pseudo-R^2^ calculations were conducted using the R packages “lme4” and “rcompanion” (version 4.2.2), respectively.

## Results

### Distribution of sociodemographic characteristics and neurocognitive performance

In the multiplex sample of 595 patients with SZ from 314 multiplex families, 223 patients with early-onset SZ (≤ 18 years of age) were younger, had a lower education level, and had lower undegraded CPT d´ than 372 patients with late-onset SZ (> 18 years of age), with no differences in terms of degraded CPT d´ and two indices of the WCST (Table 1). In the simplex sample of 595 patients with SZ matched 1:1 by sex and onset age to the multiplex sample, the early- and late-onset patients presented similar differences in sociodemographic and neurocognitive performance.

### Association of PRS for SZ with early-onset SZ

**Figure 1:**
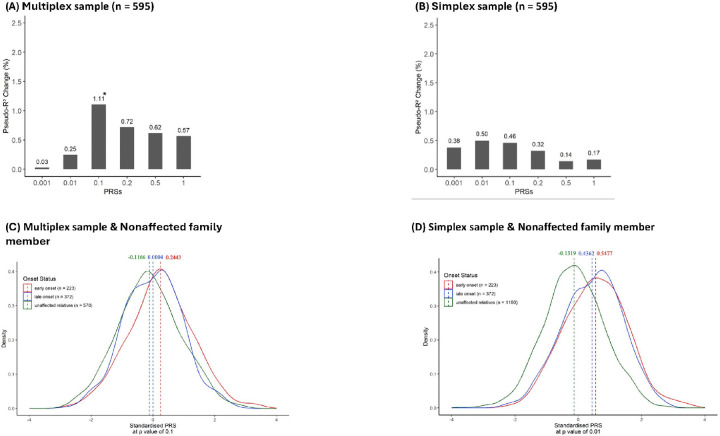
Proportion of variance in early-onset SZ explained by SZ-PRSs at various p value thresholds (x-axis), with the y-axis showing the change in Nagelkerke pseudo-R^2^ for the multiplex (A) and simplex (B) samples. Changes in R^2^ were estimated by comparing the model fit (covariates plus SZ-PRS) vs. the null model (only covariates). The mixed-effects logistic regression model, with family index as a random effect, is used in the multiplex sample, and standard logistic regression is used in the simplex sample. The significance of SZ-PRS is set at p < 0.0083 (0.05/6, Bonferroni correction). Density plots showing the best-performing SZ-PRS distributions for early-onset SZ (red), late-onset SZ (blue), and their unaffected relatives (green) in the multiplex (C) and simplex (D) samples. The SZ-PRS follows a normal distribution, scaled to a mean of 0 and a standard deviation of 1.

Regarding the extent to which the difference in onset age was attributed to genetic predisposition, the SZ-PRS at the *p*-value threshold of 0.1 was associated with early-onset SZ (*p* = 0.03) and explained the most variance of early-onset SZ (pseudo-R^2^ = 1.11%) in the multiplex sample (Fig. 1A), whereas the SZ-PRS at the *p*-value threshold of 0.01 was not associated with early-onset SZ (p = 0.14) but explained the most variance of early-onset SZ (pseudo-R^2^ = 0.50%) in the simplex sample (Fig. 1B). We then compared the SZ-PRS scores of the two groups of patients with those of unaffected relatives using the same method of PRS derivation. Among both the multiplex (Fig. 1C) and simplex (Fig. 1D) samples, patients with early-onset SZ had the highest average SZ-PRS score, followed by patients with late-onset SZ and then their unaffected relatives.

### Predicted GeRS for SZ

Before building the GeRS for our samples, we obtained a weighting system for the predicted expression of individual SNPs through TWAS of SZ in an existing database to identify the associations between the *cis*-genetic component of expression and SZ. Among the 10,358 genes included in the existing SNP-expression prediction model from the CMC (12) for the DLPFC, the expression levels of 10,306 genes identified in the GWAS of PGC SZ cohort using S-PrediXcan were successfully predicted, with the prediction performance per gene ranging from 0.8–81.5% and a mean of 8% (s.d.=11.1%). A comparison of the predicted expression levels of these genes in the PGC for patients with SZ and their controls revealed that 46 genes showed associations with SZ that reached genome-wide significance (p < 5×10^− 8^) (**Additional file 2: Supplementary Data 1**).

We then utilized the effect size of the preceding TWAS of SZ as the weight of the predicted expression of individual SNPs to derive the predicted SZ-GeRS for our samples. **Figure S3** depicts the genome-wide association between the predicted SZ-GeRS and early-onset SZ in the multiplex sample, with 7834 genes predicted to be expressed; none of them showed an association that reached genome-wide significance. When the same analysis was repeated in the simplex sample, 7987 genes were predicted to be expressed, with none reaching genome-wide significance (**Additional file 1: Figure S4**).

### Module-based GeRS for SZ and early-onset SZ

**Figure 2:**
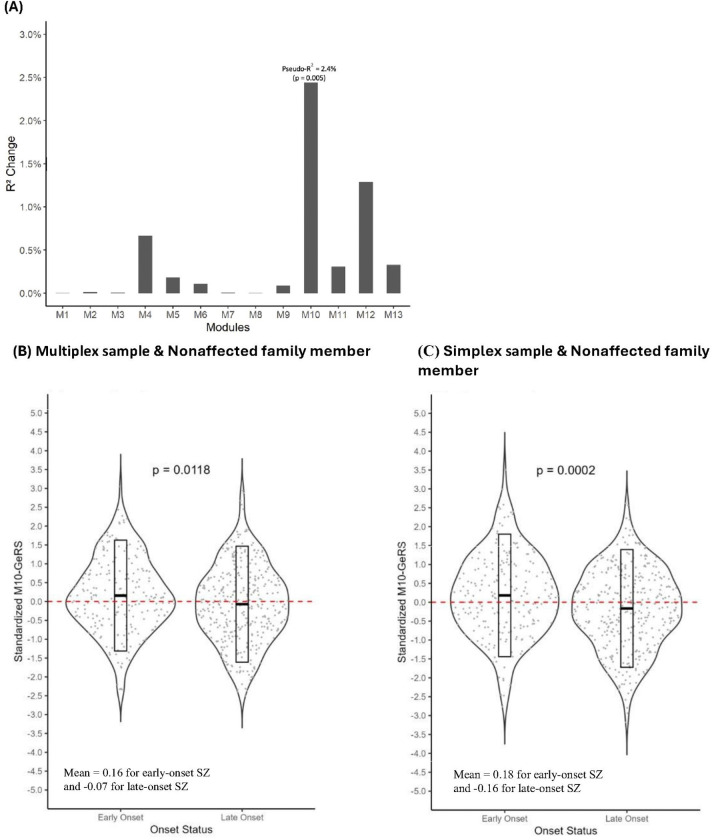
Predictive utility of individual module-based GeRS, derived from WGCNA gene lists based on cerebral cortex expression, for early-onset SZ in the multiplex sample (A). The X-axis shows modules 1 to 13, and the Y-axis indicates the change in Nagelkerke pseudo-R^2^ by comparing the model fit (covariates plus SZ-PRS) to the null model (only covariates). A mixed-effects logistic regression with family index as a random effect was used. Violin plots of M10_sub_-GeRS according to onset status for the multiplex (B) and simplex (C) samples. The M10_sub_-GeRS were based on a subset of module 10 consisting of 56 genes predicted to have genetically regulated expression in DLPFC, identified in both the multiplex and simplex samples. The M10_sub_-GeRS follows a normal distribution, scaled to a mean of 0 and a standard deviation of 1.

To capture the possible contribution of the combination of expression levels of a group of genes, we turned to 13 modules derived from coexpression analysis in a previous study using *postmortem* brain tissues of patients with five major psychiatric disorders versus those of healthy controls (25). When the association between individual module-based SZ-GeRS and early-onset SZ was assessed in the multiplex sample, the SZ-GeRS based on module 10 (M10-GeRS) explained the most variance in early-onset SZ (pseudo-R^2^ = 2.4%, p = 0.005; Fig. 2A). Among the 161 genes included in the original module 10, 94 had SNP-expression weights in the DLPFC, 57 of which were predicted to have genetically regulated expression in our multiplex sample (**Additional file 1: Table S2)**. We then examined the same 94 genes located in the DLPFC of module 10 in the simplex sample and found that 61 genes were predicted to have genetically regulated expression, with 56 of them also appearing in the multiplex samples. Hence, these 56 genes were considered a subset of M10 (M10_sub_), and the corresponding SZ-GeRS was denoted as M10_sub_-GeRS. Patients with early-onset SZ presented significantly greater standardized M10_sub_-GeRS values than did those with late-onset SZ in both the multiplex sample (Fig. 2B) and the simplex sample (Fig. 2C).

### Hub genes for early-onset SZ

**Figure 3:**
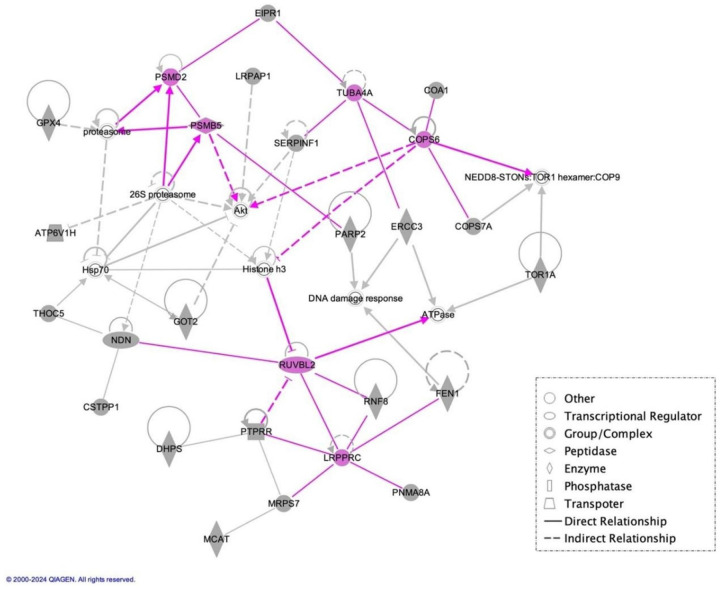
The 56 genes of M10_sub_-GeRS listed in module-10 forming the top gene network, as identified using Ingenuity Pathway Analysis (IPA). Gray shading: genes from module 10 subjected to the network analysis. Straight lines represent direct relationships between genes, whereas broken lines signify indirect relationships. Hub genes are colored in red (i.e., genes in regulatory relationships with at least 4 other genes).

To identify possible hub genes, we conducted a network analysis of the 56 genes of M10_sub_-GeRS in the multiplex sample using the IPA and revealed four networks with functionally connected genes, in which only one network had an IPA score > 40, i.e., a score of 75 for Network 1 (**Additional file 2: Supplementary Data 2)**. Focusing on this network, we identified *RUVBL2, COPS6, TUBA4A, PSMB5, PSMD2*, and *LRPPRC* as hub genes that were correlated with the expression of more than four genes within the network (Fig. 3 **and Additional file 2: Supplementary Data 3)**. When these six hub genes were used to derive M10_hub_-GeRS, the distribution of early-onset SZ tended to be greater than that of late-onset SZ in both the multiplex and simplex samples, although the difference reached statistical significance only in the latter (**Additional file 1: Figure S8)**.

### Meta-analysis of the association

**Figure 4:**
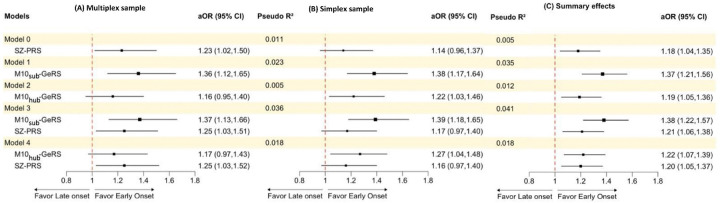
Utility of M10-GeRS and SZ-PRS for distinguishing early- from late-onset SZ was assessed in the multiplex (A) and simplex (B) samples, with summary effects (C) obtained via meta-analysis using a random-effects model. Risk scores were initially tested individually for their associations with early-onset SZ (Models 0 to 3). Advanced models incorporating gene summary GeRS and SZ-PRS data were tested in Models 3 and 4. SZ-PRS: the best-performing SZ-PRS in each sample; M10_sub_-GeRS: the gene summary GeRS based on a subset of module 10 consisting of 56 genes predicted to have genetically regulated expression in DLPFC, identified in both the multiplex and simplex samples; M10_hub_-GeRS: the gene summary GeRS based on 6 hub genes identified by the IPA top network; aOR: adjusted odds ratio from mixed-effect logistic regression with family index as a random effect in the multiplex sample and logistic regression in the simplex sample. Models adjusted for sex, education, and 4 PCs; Pseudo-R^2^: increase in R^2^ estimated by comparing the model fit to the null model (only covariates).

We then conducted a series of multivariable logistic regression analyses, adjusting for sex, educational level, and four ancestry PCs for the multiplex sample (Fig. 4A) and the simplex sample (Fig. 4B), respectively. After accounting for SZ-PRS, the variance explained by M10_sub_-GeRS (or M10_hub_-GeRS) was 2.5% (or 0.7%) in the multiplex sample and 3.6% (or 1.3%) in the simplex sample. In terms of the association of M10_sub_-GeRS (or M10_hub_-GeRS) with early-onset SZ, the corresponding adjusted ORs [aORs] in Model 3 (or Model 4) for the multiplex sample (Fig. 4A) and the simplex sample (Fig. 4B) were similar in direction and magnitude, although some did not reach statistical significance, i.e., M10_hub_-GeRS in Model 4 for the multiplex sample.

Hence, we aggregated both the multiplex and the simplex samples to conduct a meta-analysis to estimate summary effects (Fig. 4C). The meta-analysis revealed that the aORs of SZ-PRS (Model 0), M10_sub_-GeRS (Model 1), and M10_hub_-GeRS (Model 3) were significantly greater than one. Nevertheless, even after adjustment for SZ-PRS and the covariates, significant associations with early-onset SZ existed for M10_sub_-GeRS (aOR = 1.38, 95% CI = 1.22–1.57) and M10_hub_-GeRS (aOR = 1.22, 95% CI = 1.07–1.39). That is, reducing the genes in gene-summing GeRS from 56 (M10_sub_-GeRS) to 6 (M10_hub_-GeRS) did not diminish the association.

To examine the robustness of the module-based GeRS, we used another set of modules derived from 3 diagnostic groups (SZ, bipolar disorder, and autism) (38) to reconstruct individual module-based GeRS (more detail in the **Additional file 1: Supplementary Note**). Intriguingly, 2 of the 8 genes designated as hub genes using module 9 in these newly derived modules were also defined as hub genes in the originally identified early-onset-related module (i.e., module 10).

### Functional mapping of the hub genes

The known functions and previously established genetic association of the six hub genes, including *PSMB5* (39–42), *PSMD2* (43, 44), *TUBA4A* (45–47), *LRPPRC* (48, 49), *RUVBL2* (50, 51), and *COPS6* (52, 53), are summarized in Table 2. Briefly, they are mainly involved with cellular processes crucial for neurological function and implicated in various neurodevelopmental and neurodegenerative disorders, including proteasomal protein degradation, microtubule dynamics, mitochondrial function, chromatin remodeling, and protein degradation.

**Figure 5:**
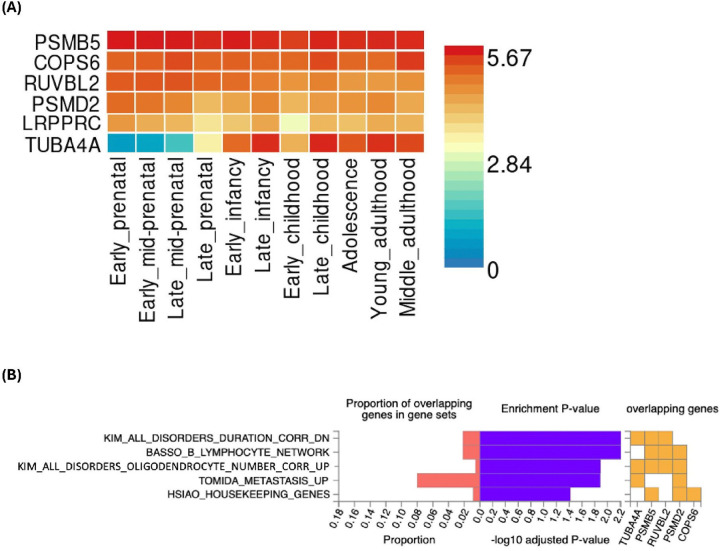
Enrichment analyses of hub genes conducted by FUMA. Gene expression heatmap for hub genes across the 11 general developmental stages of brain samples from BrainSpan, with genes ordered by expression level (A). The average expression level (log2 transformed expression value) of the genes is between 0 (blue) and 5.67 (red). Enrichment of hub genes in gene sets in FUMA using molecular signatures database (B). The −log10 adjusted P-value indicates the statistical significance of enrichment after applying the Benjamini-Hochberg (BH) correction for multiple testing. The BH method controls the false discovery rate, reducing the likelihood of false positives arising from testing multiple gene sets for enrichment.

We then performed gene-to-function analyses for those hub genes using the FUMA to understand which biological pathways or gene sets might be involved (Fig. 5). *PSMB5* and *COPS6* showed high expression from early prenatal stages through middle adulthood, while *RUVBL2* and *PSMD2* had elevated expression only during early prenatal stages, diminishing after infancy (Fig. 5A). In contrast, *TUBA4A* exhibited increased expression after infancy. Also, several chemical and genetic perturbation gene sets showed significant enrichment (Fig. 5B), including those related to psychiatric disorders duration, B lymphocytes regulatory network, and perineuronal oligodendrocytes in the BA9 brain region.

Additionally, we utilized brain single-cell expression from psychENCODE, including samples with SZ, bipolar disorder, and controls, to better understand the expression patterns of these hub genes across various cell types (**Additional file 1: Figure S9**). We found most hub genes were highly enriched in excitatory neurons compared to other cell types except *LRPPRC*.

## Discussion

Despite antipsychotics being generally used to treat the symptoms of SZ, many individuals with early-onset SZ exhibit poor responses and resistance to treatment. The antipsychotics currently available were developed based on serendipitous clinical observations of the antipsychotic properties of drugs initially used for other indications (54). Identifying new drug targets by utilizing genomic information to understand the pathophysiology of early-onset schizophrenia is essential. Here, we applied the GeRS approach to early-onset SZ and identified six hub genes, including *RUVBL2, COPS6, TUBA4A, PSMB5, PSMB2*, and *LRPPRC*. Functional mapping and enrichment analysis revealed potential biological mechanisms involved in early-onset SZ, e.g., expression changes in excitatory neurons and immune-regulatory processes. Our findings provide clues for downstream investigations into the etiology of early-onset SZ and may potentially be applied in early interventions.

Several strategies were implemented to ensure the robustness of our findings, particularly 1) examining the module-based GeRS in both the multiplex and the simplex samples of patients with SZ and then aggregating both the multiplex and the simplex samples to conduct a meta-analysis to obtain estimates of summary effects, 2) adopting the SNP-expression prediction model derived from brain tissues of patients with psychiatric disorders to avoid potential bias resulting from nontrait-related tissues in different organs (13, 55), 3) and utilizing existing brain tissue gene expression data to map the identified genes to their biological context.

Despite the differing familial loadings in the multiplex and the simplex samples of patients with SZ, the differences in neurocognitive performance and SZ-PRS between early-onset SZ and late-onset SZ remained and were in line with previous findings, e.g., early-onset SZ displaying more neurocognitive impairment (56, 57) and higher SZ-PRS (58, 59) than late-onset SZ. This, in part, justified our conduction of meta-analysis to estimate the summary effects of the identified genes.

Although none of the individual SNPs in SZ-GeRS were significantly associated with early-onset SZ in this study, similar to previous studies (15, 60), we summarized genes from empirically derived gene clusters to obtain a functionally meaningful SZ-GeRS (25, 38). The modules derived from the postmortem brain tissues of patients with five psychiatric disorders (25) led to greater variance explained by the M10_hub_-GeRS than that explained by the modules from patients with three psychiatric disorders and two specific brain regions (38). There are several possibilities, including the following: 1) many mental disorders of different diagnostic categories have shared genetic susceptibility; 2) preselection of brain regions may overlook contributions from other regions; 3) the gene list of module 10 has been found to overlap with a neuronal mitochondria gene-enriched module previously associated with fundamental neuronal processes and psychiatric disease (25); and 4) module 10 has been used to enrich the coexpression module profiling from the brain tissues of mouse models harboring SZ and autism-associated mutations, identifying its association with neuronal energetics and firing rate (61).

Our findings support the postulation that GeRS has better portability across ancestry groups for predicting traits compared to conventional PRS (62, 63), based on the hypothesis that GeRS, derived from quantitative-trait locus variants potentially shared among different ancestral groups, is associated with shared biological mechanisms. Moreover, we observed that adding module-based SZ-GeRS to the model containing SZ-PRS and potential confounders would substantially increase r^2^ and maintain the significant pattern of main effects in both the multiplex and the simplex samples. This implies that module-based SZ-GeRS and SZ-PRS each provided their respective contributions to early-onset SZ and that module-based SZ-GeRS might be a gene-based complement to SZ-PRS, potentially improving the ability to distinguish between early-onset and late-onset SZ. Our findings align with the fact that GeRS and PRS quantify independent genetic effects on complex traits (14, 62) and that integrating information from functional GeRS can improve risk prediction for these traits (63, 64).

Based on the 56 genes of one subset of M10, M10_sub_, this study utilized network analysis to narrow down to six hub genes that were predicted to highly regulate the expression of other genes within the coexpression network of module 10, and hence their aberrant expressions might impact downstream biological functions and lead to the onset of the illness (65). This possibility is further supported by subsequent explorations of the hub genes, from literature review to functional mapping of gene sets in developmental temporality and specific diseases or traits, and to single-cell gene expression profiles. These enrichment analyses provide further insight that these hub genes might have diverse expression patterns across developmental stages. Their coordination during brain development may reflect an atypical “transcriptional program” that deviates from normal development (66). This deviation could contribute to abnormal neural processes, potentially facilitating early-onset SZ. Additionally, several enriched gene sets link these genes to neurodevelopmental processes, such as the number of perineuronal oligodendrocytes in the BA9 region, and immune dysregulation, particularly in the B lymphocyte regulatory network. Reduction of perineuronal oligodendrocytes, as reported in a recent systematic review of postmortem brain studies (67), has been associated with major psychiatric disorders, and immune system dysfunction and inflammatory processes have long been associated with these disorders (68). The enriched gene set linked to the duration of psychiatric disorders might account in part for the clinical characteristics of early-onset SZ, i.e., being associated with more severe symptoms and treatment resistance, and hence resulting in a longer duration of illness. Moreover, most hub genes were enriched in excitatory neurons, indicating these cell types are likely central to the relationship between gene expression and psychiatric disorders. This support recent idea (69) that genetic risk variants affect genes with measurable expression changes in excitatory neurons of individuals with SZ.

These hub genes have significant clinical relevance due to their dual roles as both susceptibility genes and modifier genes for SZ. While most antipsychotics are designed to target susceptibility genes, early-onset SZ involves modifier genes. This distinction may explain why patients with early-onset SZ often exhibit poor responses to existing antipsychotics, resulting in treatment resistance. Hence, our findings provide valuable insights for identifying potential drug targets and improving treatment for the earliest stages of this illness.

Our study has several limitations. First, as this study was based on a case-only design, we potentially identified only the ‘mixed genes’ for SZ, which have dual functions as susceptibility genes and modifier genes (23, 70), but overlooked purely modifier genes for early-onset SZ. Second, the small sample size of this study might render individual SNPs included in M10_sub_-GeRS and M10_hub_-GeRS not being able to reach transcriptome-wide significance. Nevertheless, the combination of these genes was able to show strong associations with early-onset SZ. Last, the aberrant gene expression, as incorporated in the module-derived GeRS, was based on prediction; future *in vitro* and animal studies are needed to elucidate their roles in the biological mechanisms of early-onset SZ.

In conclusion, we found that a module-based GeRS approach that incorporates predicted gene expression resulting from the interaction of genes and environmental factors may help capture further contributions to early-onset SZ. Pathway and enrichment analyses revealed that the identified hub genes might be involved in deviation from typical brain development and expression changes in excitatory neurons. Our findings will enhance the understanding of SZ etiology and may contribute to early screening and personalized prevention efforts.

## Figures and Tables

**Table 1:** Distributions of sociodemographic characteristics and neurocognitive performance indices in patients with schizophrenia grouped by early- and late-onset in the multiplex and simplex samples, respectively.

	Multiplex sample					Simplex sample				
Variables	Early-onset patients^a^ (N = 223)		Late-onset patients^a^ (N = 372)		Group Comparison^b^	Early-onset patients (N = 223)		Late-onset patients (N = 372)		Group Comparison^c^
	Mean	(S.D.)	Mean	(S.D.)	p	Mean	(S.D.)	Mean	(S.D.)	p
Male sex, n %	131	(58.7%)	224	(60.2%)	0.72	131	(58.7%)	224	(60.2%)	0.72
Age, years	30.72	(7.69)	34.63	(6.72)	<0.001**	30.72	(7.61)	35.94	(7.47)	< 0.001**
Age at onset, years	15.99	(2.13)	24.17	(5.09)	< 0.001**	16.08	(1.89)	24.03	(4.95)	< 0.001**
Education level, years	9.68	(2.49)	11.07	(2.83)	< 0.001**	11.41	(2.40)	12.21	(2.48)	0.001**
CPT indices^d^										
Undegraded CPT d´	−2.74	(2.15)	−2.26	(2.03)	0.018*	−2.16	(2.07)	−1.70	(1.77)	0.019*
Degraded CPT d´	−2.56	(1.69)	−2.51	(1.47)	0.782	−1.46	(1.66)	−1.27	(1.71)	0.276
WCST indices^e^										
Categories achieved	−1.09	(0.72)	−1.04	(0.84)	0.476	−0.90	(0.98)	−0.88	(0.96)	0.882
Perseverative errors	1.23	(1.74)	1.39	(1.82)	0.361	1.15	(1.70)	1.28	(1.72)	0.457

* p value < .05;

** <0.01

a 106 individuals in the early-onset group (47.5%) and 250 individuals in the late-onset group (67.2%) were siblings from multiplex families with SZ.

b The group comparisons were conducted using a mixed-effect logistic regression model with family index as a random effect.

c The group comparisons were conducted using logistic regression.

d The adjusted z scores were derived by means of standardizing the raw scores with adjustments for sex, age and education against a community sample of 345 individuals (31).

e The adjusted z scores were derived by means of standardizing the raw scores with adjustments for sex, age and education against a group of 392 healthy controls (32).

**Table 2:** Background on the six hub genes identified from module 10, including brief descriptions of their genetic functions and previously established genetic associations.

Gene	Known genetic functions and relationships
*PSMB5*	*PSMB5* encodes the proteasome subunit beta type-5. The proteasome system is involved in regulating major cellular pathways, including those leading to inflammation (39), and has been identified as a canonical pathway associated with SZ (40, 41) and treatment resistance in major depressive disorder (42).
*PSMD2*	*PSMD2*, which encodes a non-ATPase subunit of the 26S proteasome, is crucial for regulating protein degradation and cellular processes (43). Recent in vivo studies show that neuronal membrane proteasomes regulate neuronal circuit activity, potentially involving PSMD2, and learning-induced behavioral plasticity, thereby highlighting the role of PSMD2 in neuronal function (44).
*TUBA4A*	*TUBA4A* encodes alpha tubulin 4A, a key component of microtubules regulating axonal transport in the central nervous system (45). *TUBA4A* demonstrates ubiquitous expression across all cell types, with its highest levels observed in the brain (46), and has been associated with amyotrophic lateral sclerosis and frontotemporal dementia (47).
*LRPPRC*	*LRPPRC*, which encodes leucine-rich pentatricopeptide repeat-containing protein, plays a crucial role in mitochondrial function and is involved in regulating mRNA stability and polyadenylation and coordinating mitochondrial translation (48). LRPPRC deficiency is linked with ATP synthase deficiency and severe neurodegenerative disorders such as Leigh syndrome, underscoring its critical role in brain function (49).
*RUVBL2*	*RUVBL2* encodes RuvBL2 (RuvB-like 2), which is part of various ATP-dependent chromatin remodeling complexes that are involved in the regulation of gene expression (50). *RUVBL2* predominantly manifests in the SOX2-positive compartment of cerebral organoids and mid-gestation fetal brain tissue, with its chemical inactivation leading to precursor cell displacement and apoptosis and genetic variants linked to neurodevelopmental impairments (51).
*COPS6*	*COPS6* encodes the sixth subunit of the photomorphogenic 9 (COP9) signalsome (CSN), which is involved in the ubiquitin-mediated protein degradation process (52) and is further implicated in neurodegenerative diseases (53).

## Data Availability

The datasets used and analyzed in the current study are not publicly available due to conditions in the participant consent and other ethical restrictions. However, the data that support the findings of this study are available from the corresponding author upon reasonable request.
